# Impact of Daily Choral Singing and Creative Writing Activities on the Cognitive Development of Second-, Third-, and Fourth-Grade French Children from Low Socioeconomic Backgrounds

**DOI:** 10.3390/children10091515

**Published:** 2023-09-06

**Authors:** Angélica Gutiérrez Cisneros, Juliette Roussey, Talya Inbar, Althea Fratacci, Aline Frey

**Affiliations:** 1 Laboratoire de Neurosciences Cognitives (LNC), CNRS, Aix-Marseille University, 13003 Marseille, France; angelica.gutierrez-cisneros@etu.univ-amu.fr (A.G.C.); juliette.roussey@etu.univ-amu.fr (J.R.); talya.inbar@etu.univ-amu.fr (T.I.); 2 Laboratoire de Psychologie et NeuroCognition, UMR CNRS 5105, Université Grenoble Alpes, CEDEX 9, 38058 Grenoble, France; althea.fratacci@univ-grenoble-alpes.fr

**Keywords:** children development, choral singing, creative writing, cognitive abilities, transfer of learning

## Abstract

In France, around one-fifth of children have reading difficulties, and school results are highly dependent on their socio-economic status. In this context, the need for alternative and innovative teaching techniques holds importance, and more artistic approaches are promising. The aim of this study was to assess the impact of a daily choral singing or creative writing practice on the cognitive and linguistic development of French children from disadvantaged backgrounds. Eighty children participated in this longitudinal study, for whom we measured several cognitive and linguistic skills at the beginning (pre-test) and end (post-test) of the school year. The results showed that children in “singing” classes improved both their reading skills and processing speed, while those in “writing” classes improved their reading skills and vocabulary. These results open up new avenues of learning support, specifically for children with difficulties.

## 1. Introduction

The development of the five fundamental learning skills—reading, writing, arithmetic, reasoning, and cooperation—does not occur naturally, but comes as the result of pedagogical approaches and is of utmost priority during the first years of schooling [1]. However, even with teaching designed to develop these skills, it is shown that on average, nearly 20% of French children have reading difficulties [2]. Moreover, OECD ranks France as one of the countries with the strongest correlation between student achievement and socio-economic status. Thus, French pupils from disadvantaged backgrounds obtain poorer results on average than those from advantaged backgrounds and are five times more likely to be in difficulty at school. For example, the writing level of the 10% of students from the wealthiest families is about three school years ahead of that of the poorest 10% of students [3].

Thus, despite the scientific advances in fields such as the psychology of learning and pedagogy, teachers often remain powerless to remedy the difficulties of their pupils. Faced with this observation, new alternatives appear to be indispensable [4]. Accordingly, the objective of this study is to test the effects of a daily, in-school choral singing and creative writing practice on the cognitive and linguistic development of children.

### 1.1. Choral Singing

The body of the literature regarding the effects of music training on cognitive skills, brain functions, and/or academic achievements of children has grown considerably in recent years. However, the effects of this practice are still debated, and recent meta-analyses have attempted to shed some light on this issue [5,6,7,8]. Overall, studies show small but significant effects of musical training, which vary widely depending on the quality of the experimental design (e.g., presence or absence of active control groups, random allocation of participants), the definition of the term “musical training” (e.g., amount of instrumental practice), and the way in which near and far learning transfers are differentiated.

In these different studies, music education aims to promote musical skills, such as rhythm, basic music notation, and pitch and tone discrimination, and in most cases, involves learning an instrument. Singing is sometimes included in musical training, but a clear methodological distinction is not always made between instrumental and singing practice. To our knowledge, very few studies (at least fewer than the study of instrumental practice) have looked at the effects of singing per se. Experiments using brain imaging methods revealed interactions between the linguistic and melodic dimensions of sung words, as well as the activation of common neural networks in the perception of spoken words, vocalization (singing without words), and sung words [9,10]. These results paved the way for the hypothesis that if the processing of spoken and sung words involves common interactive processes and brain regions, then singing training could improve speech perception and production as well as general cognitive abilities involved in language. In line with this hypothesis, some studies have looked at the use of singing in language development, specifically on phonological awareness, and have shown improvement in these skills compared to control groups using more traditional learning methods (that is, without the systematic use of singing) [11,12]. More specifically, singing has been shown to promote vocal and motor flexibility and improve speech imitation (particularly phonetic language skills) and auditory working memory in adults [13,14], 9- and 10-year-olds [15], and preschoolers [16]. Similarly, Degé et al. found that song learning abilities were significantly correlated with phonological awareness abilities in 9-to-12-year-olds, and more specifically, that song learning abilities were a good predictor of phonological awareness [17]. Finally, the developing literature has shown the effects of choral singing on pro-social skills. Indeed, group singing generally requires a high level of cooperation between individuals, as well as the need to synchronize (synchronization observed, for example, at the level of laryngeal muscles, cf. [18]; or respiratory patters, cf. [19]). Movement synchronization appears to influence interpersonal affiliation and the ability of groups to form social bonds [20].

However, these studies have always used a cross-sectional approach (i.e., comparing the skills of singers vs. non-singers at a point in time), and correlation is not causation [21,22]. Thus, the only way to accurately determine whether singing practice can mediate reading acquisition or more general cognitive skills is through longitudinal studies. Here, we aim to do just that, by comparing three groups of children (none of whom had singing training prior to this experiment) over the course of one school year. The first group participated in daily choral singing activities, the second in daily creative writing workshops, and the third performed no specific activity, and served as a control group.

### 1.2. Creative Writing

There has been a great deal of research on creativity since about the 1950s, and many definitions have been attributed to this concept. Recently, a consensus has emerged with a standard “bipartite” definition of creativity as requiring both originality and effectiveness [23]. The former term refers to the uniqueness and novelty value of a proposition, while the latter refers to its relevance, appropriateness, and utility [24]. In other words, creativity requires the ability to conceive ideas and generate responses that are not only novel, but also adaptive [25].

Until recently, creativity was often confused with disobedience in school settings, as students were expected to act passively and receive instructions as prior generations did [26]. For example, Lucas and Greany asked students what activities occurred most frequently in their classrooms [27]. The three most frequently mentioned activities were: copying from the board or a book (56%), listening to a teacher talk for a long time (37%), and having a class discussion (37%). Fortunately, priorities have shifted since then, and especially since 2009; then the OECD published a report on the new millennium skills for learners in the context of the “European Year of Creativity and Innovation”. Creativity was identified as one of the key skills for the 21st century, given its importance in determining individual and social outcomes [28]. However, while creativity has been valued, it is still not considered a skill that can be developed in schools, and few teacher training programs focus on developing this skill [29]. On the other hand, some studies show that creativity has had little or no association with academic achievement (e.g., [30,31,32]). Overall, the results of the research illustrate that the correlation values between creativity and school achievement are highly diverse and range from negative correlations (e.g., r = −0.07; [33]) to strong positive ones (e.g., r = 0.66; [34]). There are also important differences in cultural and educational systems [35]. Authors have shown that as the socioeconomic level increases, so does the creative ability [36], as individuals with a higher socioeconomic status (SES) often have more learning resources and opportunities. Further research notes that the impact of creative thinking on academic performance is inconsistent across student demographics and plays a more positive role in the academic performance of boys and children from disadvantaged backgrounds [37]. Thus, although creativity appears to be an important individual characteristic, its potential role in academic learning is not yet clearly established.

In our study, we specifically test the possible impact of creative writing workshops on the development of cognitive and language skills. The most common definition of creative writing is one that produces narratives, stories, plays, or poems, as opposed to more formal writing [38]. The most important aim of creative writing activities is to help students express their feelings and thoughts in an original, fluent, and clear way, instead of writing repetitive and monotonous texts [39]. Pirolu defines creative writing as a practice that aims to develop sensory perception, imagination and observation, fictional expression, and critical thinking skills. It should also help authors break down prejudices to find a language that is uniquely their own. Furthermore, creative writing can serve to nurture the natural interest in writing and an inherent need for expression that many children enter school with [40]. Indeed, others agree that creative writing provides entertainment, encourages artistic expression, stimulates the imagination, and allows the various functions of writing to be explored [41].

Thus, the objective of our research is to compare the effects of two artistic practices, choral singing and creative writing, as well as to compare both of these practices with a control group, to quantify differences between them and a developmental baseline. As explained earlier, both activities involve and develop specific and common elements. Regarding the brain bases of these two activities, while singing is supported by the activation of the motor and auditory cortical areas of the brain [42], creativity has been linked to frontal lobe activity, suggesting that it shares the same neural circuits associated with executive functions [43]. Therefore, we hypothesize that singing could improve memory and attention, as it has been shown with musical practice, as well as oral language (phonological awareness, reading). Furthermore, although the literature on this subject is still sparse, we believe that singing could also improve pro-social skills. On the other hand, we expect creative writing workshops to specifically improve creativity, as well as oral and written language (graphomotricity, word dictation). Finally, we expect that the other abilities tested (non-verbal intelligence, speed processing) will be improved in the same way by the two workshops, and will have a greater improvement than the control group not carrying out any of the workshops.

A strong point of this study is that the two workshops take place each morning at school, contrary to previous studies where workshops often occur only once a week, at best. This frequency is important, as the benefits of repetition in learning have been well established [44], and the neuroanatomical and functional effects of musical practice are observed when the practice is done a minimum of three times a week [45]. Here, singing and writing workshops had the same duration, and leaders discussed with each other to ensure a similar progression, thus guaranteeing a similar level of motivation and commitment from students. Moreover, our research focuses on children from disadvantaged backgrounds, for whom we have seen the greatest effects of music and creativity practices [46]. Finally, our research is based on a longitudinal protocol, measuring the children’s skills before and after the different workshops, thus ensuring the causality of the observed effects.

## 2. Materials and Methods

### 2.1. Participants and Procedure

Eighty French children (34 girls, 46 boys) aged from 7 to 10 years old, from a primary school located in the northern districts of Marseille characterized by high levels of poverty and unemployment (IPS score = 91.5, DEEP, 2021–2022), participated in this experiment (see Appendix A: Table with the characteristics of the children). They came from seven classes of three different grades: three 2nd-grade classes, two 4th-grade classes, and two 5th-grade classes. For each grade, one class attended the choral singing activity and the other creative writing; for the 2nd grade, a third class did not do any particular activity and served as the passive control group, allowing for a measure of development that was independent of any specific practice. There was thus a 2nd-grade CG (Control Group), a 2nd-grade SG (Singing Group), a 2nd-grade WG (Writing Group), a 4th-grade SG, a 4th-grade WG, a 5th-grade SG, and a 5th-grade WG. All of the students in these groups (except the control group) participated in the daily activities (singing or writing), which were integrated in the school’s curriculum. However, for practical reasons (mainly due to the COVID-19 pandemic), only a subset of the children was tested as part of the scientific project. Those children were randomly selected such that the distribution of girls and boys in each group and their average academic capacities, as determined by their teachers, was roughly even. Overall, 16 children from each of the 2nd-grade classes, and 8 from the 4th-grade and 5th-grade classes participated in the scientific study. We chose to test more 2nd graders as the effects of singing and creative writing practice are expected to be greater the younger children are, and because we had a passive control group for this grade [6]. All participants had normal or corrected vision and normal hearing.

This study was conducted in agreement with guidelines for the protection of human participants as defined in the declaration of Helsinki and was approved by the local ethics committee of Aix-Marseille University (N/Ref: 2022-11-24-006). All parents gave their informed written consent for their children to participate in the study. The procedure was carefully explained to the children to ensure that they agreed to participate in individual testing sessions in a quiet classroom at the school, and they were informed that they could stop participating in the tests at any time.

### 2.2. Study Design

All the children in the six groups received either singing (SG) or creative writing workshops (WG), every morning (thus four times a week, as the children did not have school on Wednesdays), for 20 min each morning, while the 2nd-grade control class followed the usual school curriculum (CG).

The children tested in the longitudinal scientific study participated in a series of pre-tests, between mid-September and mid-October 2020, and then repeated these measures as post-tests, from mid-April to mid-May 2021.

### 2.3. Choral Singing and Creative Writing Activities

The singing activities were organized in partnership with Musicatreize (https://www.musicatreize.org/), a Marseille-based choral singing association, which hired a professional singer to lead the sessions each morning. During each workshop, the children were first asked to warm up their bodies for singing to achieve a well-balanced and tension-free posture. The instructor then provided vocal warm-up exercises. Finally, the children learned songs by ear, which they practiced in polyphony and/or canon.

The creative writing workshops were organized by a professional writer from the La Marelle (https://www.la-marelle.org/) writing organization. These workshops focused mainly on writing content rather than its form (for example, spelling mistakes were not corrected), as the main objective was for students to use their imaginations and creativity. Thus, children were asked to write short texts of their own invention, following a prompt, such as to create a dialogue between inanimate objects of their choice. Each proposed theme was first discussed and explored collectively, then during the following sessions the children were asked to write, and finally volunteers were chosen to read their text in front of the class, which was then discussed as a group.

Instructors worked together to design the two workshops, so as to ensure a similar progression in both, and to generate the same degree of motivation and commitment among the children. In addition, students in the workshops worked towards a final project, which was to set some of the students’ texts to music to be sung at an end-of-year concert. The children’s texts were also compiled in a small booklet that they were able to take home at the end of the year.

### 2.4. Materials and Measures

#### 2.4.1. Singing and Creative Writing Assessment

To assess the improvement of the children’s singing or creative writing skills in each of the workshops, two external juries were set up, one consisting of professional singers and the other of professional writers. These two juries assessed the children during pre- and post-test periods.

Each judge assessed the children individually according to a rubric consisting of six scored criteria on a scale of 0 to 5. For the singing workshops, the criteria were the ability to sing spontaneously, the level of focus (posture and ability maintain attention), the ability to sing together in unison and in polyphony, rhythmic accuracy, intonation accuracy, and vocal delivery. For creative writing, the students were judged based on their starting speed, capacity for collaboration, flexibility, imagination, freedom of expression, and ability to follow a given writing style.

#### 2.4.2. Psychometric Tests

The Evaluation of Children’s Creative Potential Test (EPoC [47], see Appendix B) was chosen to assess students’ verbal and graphical creativity, based on its 2 sub-components: divergent thinking (generating several options from a given starting point) and convergent thinking (proposing an integrative solution from a group of ideas or criteria). Thus, 4 subtests were presented to the children, assessing divergent graphical, convergent graphical, divergent verbal, and convergent verbal creativity. For each of these 4 subtests, the children had five minutes to produce their work.

**Figure A1:**
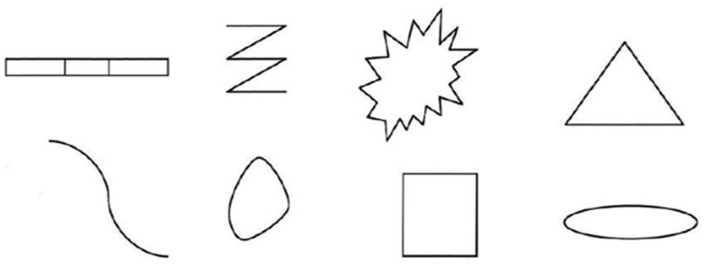
Imposed shapes in convergent graphic test.

**Figure A2:**
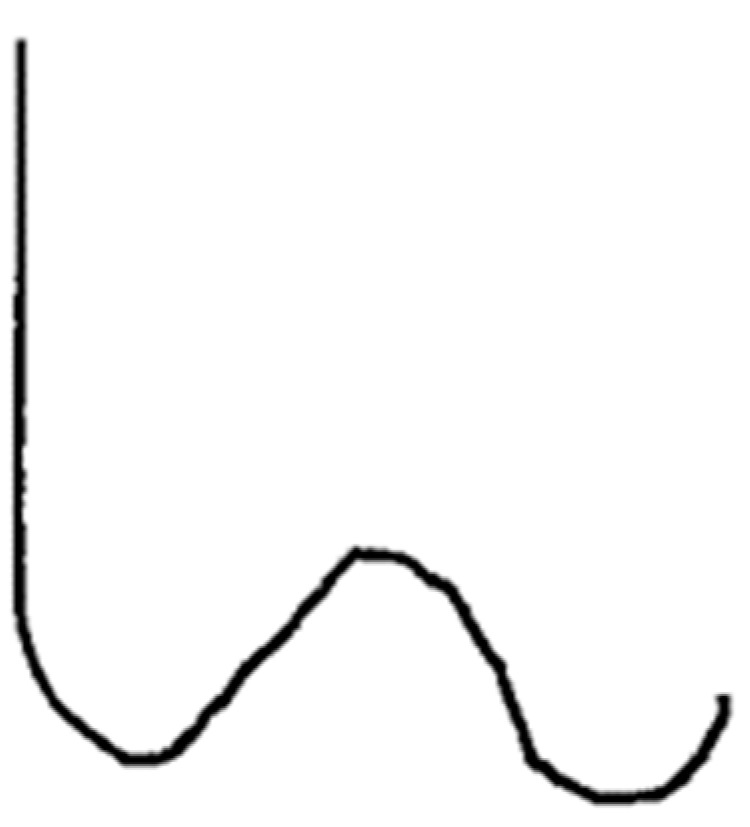
Imposed shape in divergent graphic test.

**Figure A3:**
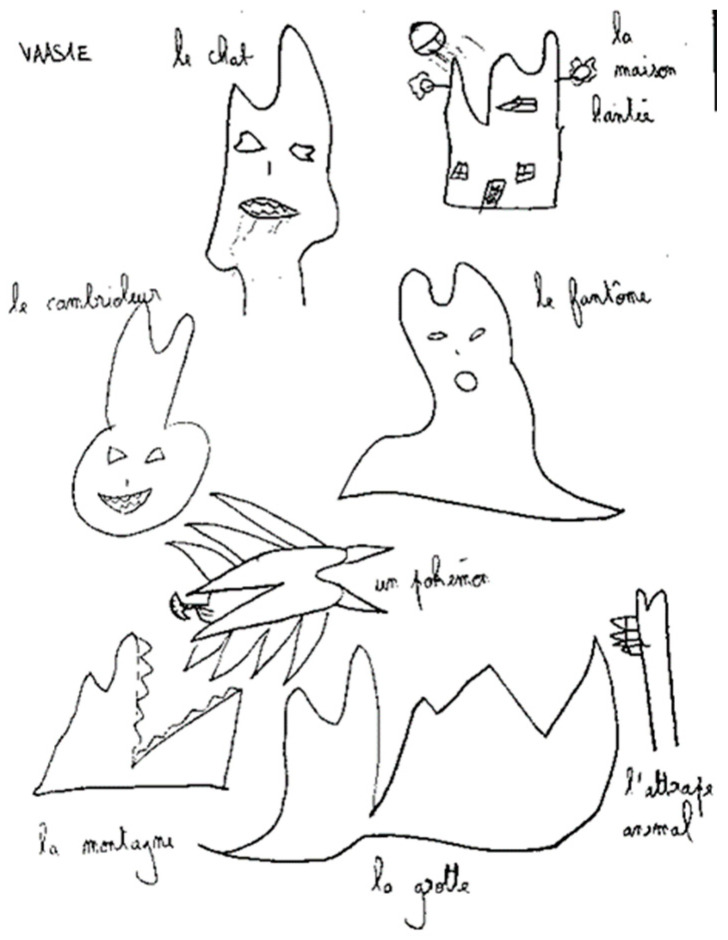
Example of a graphical divergent production.

In the divergent verbal creativity subtest, the students were asked to generate different endings to a story that was read to them, while in the convergent verbal creativity subtest, they were asked to create an original story from a given title. In divergent graphic creativity subtest, the children were presented with a small abstract shape, and is asked to draw as many original drawings as possible that included this shape. Finally, to test convergent graphic creativity, the students were presented with eight abstract shapes and were instructed to produce a single original drawing that included at least four of these shapes (see Figure A1 and Figure A2: Imposed shapes in convergent and divergent graphic tests). The convergent productions were assessed on their originality and the divergent pro-ductions on their fluidity (see Figure A3 for a child production of a graphical diver-gent task). In order to complete the divergent evaluation score, a quality criterion was added, in which the evaluator assessed the complexity of the participants’ productions.

The convergent productions were assessed on their originality and the divergent productions on their fluidity. In order to complete the divergent evaluation score, a quality criterion was added, in which the evaluator assessed the complexity of the participants’ productions.

Musical aptitudes were tested by asking children to judge whether 18 pairs of piano melodies were the same or different in terms of melody or rhythm (as adapted from the Montreal Battery of Amusia-MBEA, [48]). The children listened to the pairs of melodies on headphones and indicated their responses orally to the experimenter.

To measure auditory selective attention, the Auditory Attention and Response Set Test was used (NEPSY-II battery, [49]). A paper with four colored circles (yellow, red, blue, and black) was presented to children as they listened to a recorded list of words that included targets and distractors. In the Auditory Attention sub-test, the students pointed to the red circle as quickly as possible when they heard the word “red”, and did nothing when they heard the other words. In the Response Set sub-test, the students were told to point to the red circle when they heard the word “yellow”, to point to the blue circle when they heard the word “blue”, and to do nothing when they heard the other words.

In the Verbal Fluency Test [50], children were given one minute to list as many words as possible that either began with a specific letter or belonged to a certain category (e.g., animals).

The Similarities Test (WISC-IV, [51]) was used to measure verbal reasoning and concept formation, and consisted of asking students to identify the commonality between two words (e.g., how are a butterfly and a bee similar?).

Non-verbal reasoning and logic were evaluated with the Raven’s Matrices Test [52]. In this test, the children choose from a set of 6 images to best complete a presented pattern.

The evaluation of phonological awareness and other phonological abilities such as phoneme and syllable perception, discrimination, and manipulation was performed using the Syllable Elision and Rhyme Discrimination Test [53]. This test consisted of 4 sub-tasks in which the children were asked to segment a word by syllables, to remove a specific syllable and pronounce the result, to determine if two words rhymed, and to identify the phoneme at the beginning of a given word.

Children were also asked to read one list of 20 irregular words (e.g., in French “galop”), one of 20 regular words (e.g., “dorade”), and one of 20 pseudo-words (“gavin”) aloud. These lists came from the ODEDYS Test [54]. For each of these 3 lists, the number of errors and the time taken to read the list were measured.

The Alouette Reading Test [55] was also administered, which consisted of asking students to read, in 3 min maximum, a meaningless text. The number of words read and the number of errors made in three minutes were counted.

In order to evaluate social cognition, and more specifically empathy and the theory of mind, the Detection of Faux Pas Test [56] was used. In this test, the children were read a short story, and were asked to identify if something socially inadequate occurred.

In the Digit Span Test (WISC-IV, [51]), the students heard an increasing series of digits, presented orally at the rate of one digit per second, and were then asked to repeat them forwards or backwards.

In the Letter-number Sequencing Test (WISC-IV, [51]), participants had to memorize an increasing series of letters and numbers presented orally, which they were asked to repeat by putting the letters first, in alphabetical order, and then the numbers, in ascending order (e.g., if the experimenter said “7-C-9-E-L”, the child would say “C-E-L-7-9”).

To evaluate visual attention, the D2R Test [57] was used. In this test, students were presented with several lines of the letters “d” and “p” surrounded by one to four dashes, and they had to cross out all the d’s that were surrounded by 2 dashes. The experimenter started a stopwatch and asked the students to move to the next line of letters every 20 s (even if they had not finished the line). The number of well-identified targets and false alarms were counted.

The Symbol Search Test (WISC-IV, [51]) was used to measure visual processing speed. The children were presented with rows of symbols, where each row had two target symbols on its left and five symbols to its right side. Children were asked to decide, as quickly and as accurately as possible, whether one of the left target symbols was present or not within the five target symbols on the right. The children had 3 min to complete as many lines as possible.

Hand-eye coordination and motor skills were evaluated using the Visual-motor Precision Test (NEPSY-II, [49]). A sheet of paper picturing a winding road with borders on each side was presented to the students, who were instructed to draw the trajectory of a car or bike in a single line trying not to lift the pen or go beyond the marked boundaries.

The BHK Test [58] was used to evaluate writing ability, both quantitative and qualitatively. The students were given five minutes to copy a small given text (7 paragraphs of about 5 lines each), with the instruction to write with their usual speed and handwriting. The quality of the writing was measured using criteria evaluating morphokinetic and topokinetic aspects. The production speed was evaluated based on the number of characters produced during the five minutes of the task.

The Dictation Test (ODEDYS test, [54]) was used to measure grapheme-phoneme correspondence. Children had to write down three lists of 10 words: regular (e.g., “frite”), irregular (“seconde”), and pseudo-words (“datoir”). They were evaluated based on the number of correctly written words.

### 2.5. Data Processing and Statistical Analyses

The degree of agreement between jury members for both choral singing and creative writing productions was tested using a weighted Cohen’s Kappa. The improvement (between pre- and post-tests) in the jury criteria was tested using a Student *t*-test for the normally distributed dataset, and a Wilcoxon test otherwise.

Data from the various psychometric tests were analyzed using a Repeated Measures Analysis of Variance (ANOVA), including group (SG vs. WG vs. CG) as the between-subjects factor, and session (pre vs. post) as the within-subject factor. The ANOVA was performed twice to maintain a consistent number of participants per group. Thus, either the three singing groups (2nd-grade SG, 4th-grade SG, 5th-grade SG) were compared to the three writing groups (2nd-grade WG, 4th-grade WG, 5th-grade WG), or the three 2nd-grade groups were compared to one another (2nd-grade SG vs. 2nd-grade WG vs. 2nd-grade CG). Subsequently, Tukey post hoc tests (reducing the probability of Type I errors) were used to determine the source of significant interactions. We also reported eta squared (η2) as a measure of effect size commonly used in ANOVA models, with the following rules of interpretation: 0.01: small effect size; 0.06: medium effect; 0.14 or more: large effect. All of these analyses were performed using the JASP open-source program [59].

A k-mean clustering algorithm was also implemented using an amelioration index (post-performance minus pre-performance) for the tests showing a significant pre-post by group interaction. This was done using the statistical spreadsheet “JAMOVI” [60], with an interactive partitioning (K-means), which minimizes the within-cluster variability and maximizes the between-cluster variability. The algorithm locates inherent similarities in the data items and groups them in a cluster based on their shared characteristics. This enabled the distribution of the participants into two subgroups according to their improvement level (high or low), regardless of their group or their initial level. Finally, a chi-square analysis was performed to compare the difference between the distributions given by the cluster and a theoretical distribution which assumed that the activities had no effect.

## 3. Results

### 3.1. Evaluation of Singing and Writing Abilities before and after Training

To examine the inter-rater reliability between the four different jury members, Cohen’s kappa tests were computed, showing moderate reliability between the judges’ ratings in the singing post-tests (average Kappa = 0.57), the writing pre-tests (average Kappa = 0.49), and the writing post-tests (average Kappa = 0.52) and low reliability in the singing pre-tests (average Kappa = 0.23).

In the singing group, the jury members evaluated children based on six criteria. For logistical reasons (the class was absent on the day of the evaluation), the 2nd-grade singing group could not be evaluated through post-tests. Thus, the results of the singing criteria focus only on the 4th and 5th graders. The judges’ ratings were higher after the training for three of the criteria: level of focus (t(15) = −2.584, *p* = 0.010), the ability to sing in unison and in polyphony (t(15) = −5.93, *p* < 0.001), and rhythmic accuracy (t(15) = −2.528, *p* < 0.012). No significant effects were found for the other three criteria evaluated (ability to sing spontaneously, intonation accuracy, and vocal delivery).

The writing groups were also evaluated based on six criteria. Their ratings were higher after the training only for one criterion, the ability to follow a given writing style (t(25) = −8.73, *p* < 0.001). Ratings for the starting speed criterion were lower after training (t(27) = 3.239, *p* < 0.003). No significant pre- vs. post-training differences were found for the other four criteria (capacity for collaboration, flexibility, imagination, and freedom of expression).

### 3.2. Cognitive Measures

In this section, results regarding the main effects of the factors pre- vs. post-training and the group are presented first, followed by the results of the pre–post training by group interaction for each specific test. For each part, results comparing the three groups of children in the 2nd grade (SG vs. WG vs. CG) are presented first, followed by results for children in the SG and WG from each grade (2nd, 4th, and 5th).

#### 3.2.1. Pre–Post Comparisons: Main Effect

Second-grade students only (SG vs. WG vs. CG): the main effect of the pre–post factor was significant for all tests (for most of the tests *p* < 0.001; Alouette Reading Test: *p* = 0.014; Raven’s Matrices Test: *p* = 0.003; Auditory Attention and Response Set Test: *p* = 0.014), except for the Detection of Faux Pas Test (*p* = 0.43), the EPoC Test (*p* = 0.64), and the melodic judgment subtest (*p* = 0.24).

All students (SG vs. WG): the main effect of the pre–post factor was also significant for all tests (for most of the tests *p* < 0.001; Auditory Attention and Response Set Test: *p* = 0.053; melodic criterion of the Musicality Test: *p* = 0.009) except for the Detection of Faux Pas Test (*p* = 0.12).

Overall, and in line with expectations, these results show a general improvement over the school year.

#### 3.2.2. Group Comparisons (Main Effect)

Second-grade students only (SG vs. WG vs. CG): the main effect of the group was not significant for any of the tests except for the Auditory Attention and Response Set test (*p* = 0.036), with post-hoc comparisons showing that the CG performed better than the WG (*p* = 0.043).

All students (SG vs. WG): the main effect of the group was only significant for the Similarities Test (*p* = 0.017), with the WG performing better (13.16) than the SG (11.28), and the melodic criterion of the Musicality Test (*p* = 0.035), again with the WG (10.46) performing better than the SG (9.17).

#### 3.2.3. Pre–Post by Group Interaction

##### Alouette Reading Test

**Figure 1:**
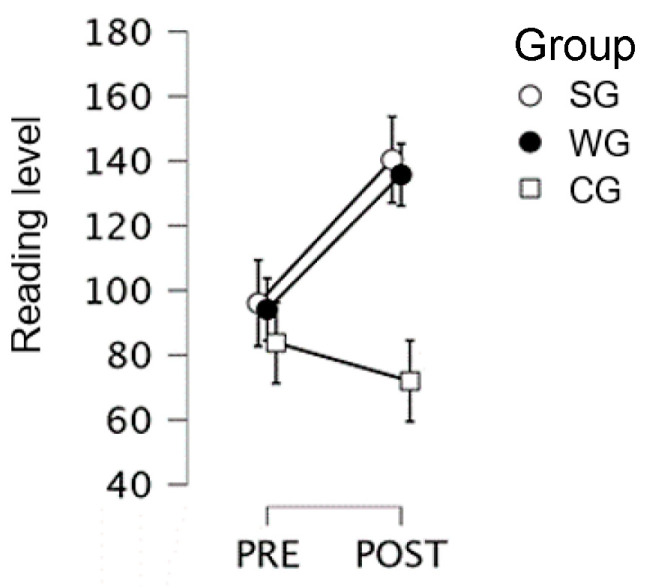
Alouette Reading Test scores for 2nd grade, for the creative Writing Group, the Singing Group, and the Control Group, at pre- and post-test.

Second-grade students only (SG vs. WG vs. CG): the pre–post by group interaction was significant (F(2, 44) = 3.6; *p* = 0.036; η2= 0.039; cf. Figure 1). Results of post-hoc comparisons showed that, after the training, the reading level was significantly higher in the WG (135.75) and the SG (140.40) than in the CG (72.00; SG vs. CG, *p* = 0.036; WG vs. CG, *p* = 0.054), with no significant between-group difference before training (SG = 96.10; WG = 94.12; CG = 83.81).

All students (SG vs. WG): the pre–post by group interaction was not significant (F < 1).

##### Similarities Test

**Figure 2:**
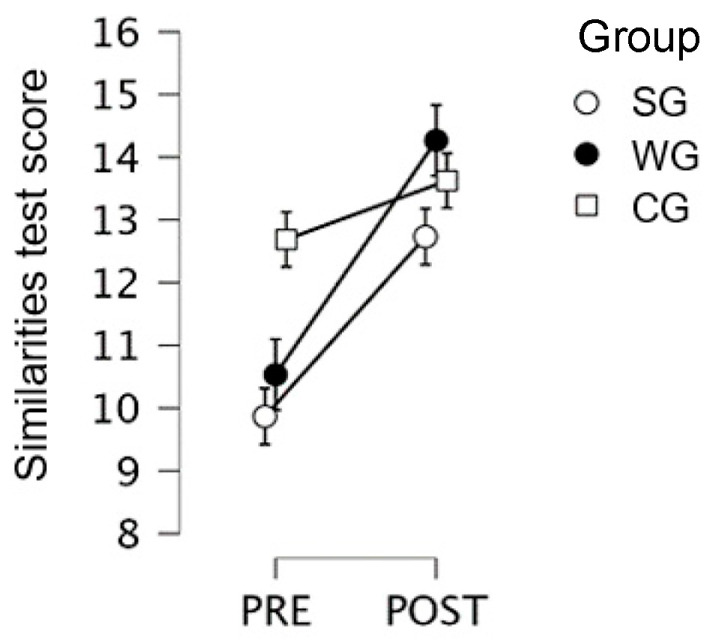
Similarities Test scores for 2nd grade, for the creative Writing Group, the Singing Group, and the Control Group, at pre- and post-test.

Second-grade students only (SG vs. WG vs. CG): the pre–post by group interaction was significant (F(2,43) = 4.46; *p* = 0.017; η2 = 0.024), with post-hoc comparisons showing a significant progression in the WG (pre = 10.53; post: 14.27; *p* < 0.001) as well as in the SG (pre = 9.87; post = 12.73; *p* = 0.002) with no significant improvement in the CG (cf. Figure 2). The between-group differences were not significant at the pre-test (CG pre = 12.69).

**Figure 3:**
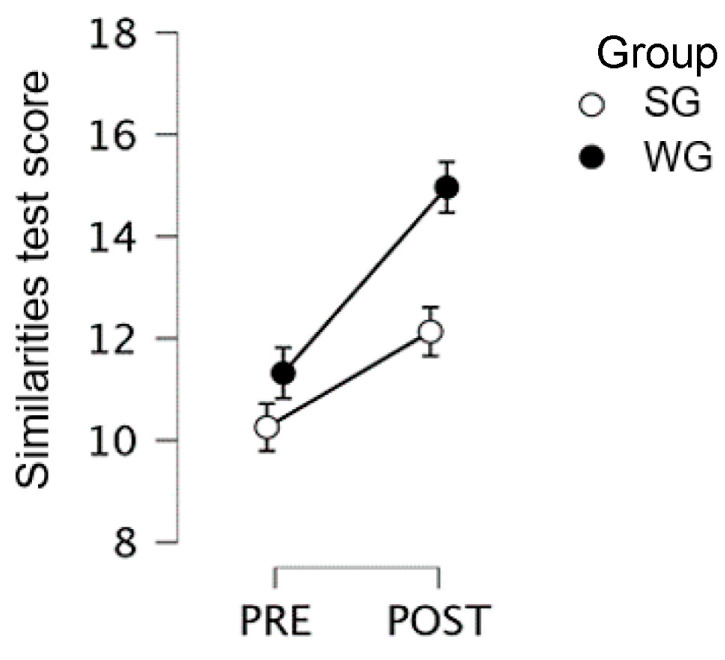
Similarities Test scores for 2nd, 4th, and 5th graders, for the creative Writing Group and the Singing Group, at pre- and post-test.

All students: the pre–post by group interaction was significant (F(1,59) = 3.84; *p* = 0.055; η2 = 0.015), with significant improvement in the WG only (pre = 11.32; post = 14.97; *p* < 0.001), as observed in Figure 3. The between-group difference at pre-test was not significant (SG pre = 10.26).

##### Symbol Search Test

**Figure 4:**
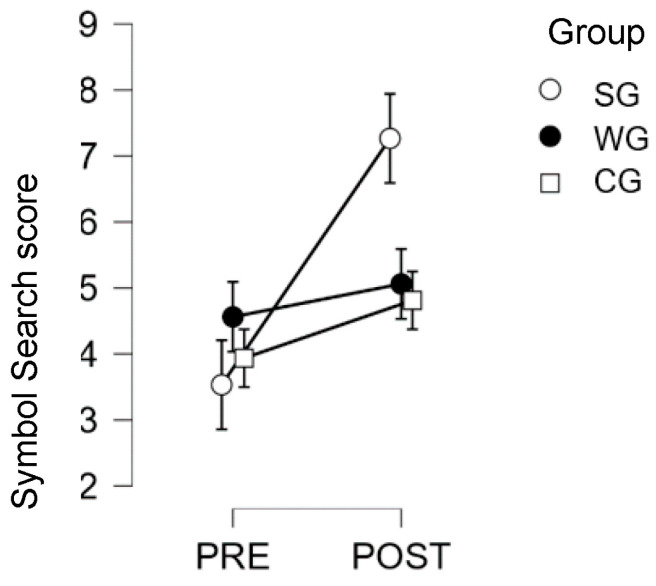
Symbol Search Test results for 2nd graders, for the creative Writing Group, the Singing Group, and the Control Group, at pre- and post-test.

Second-grade students only (SG vs. WG vs. CG): results showed a significant pre–post by group interaction (F (2,44) = 5.06; *p* = 0.011; η2 = 0.056), with significant improvement only in the SG (pre = 3.53; post = 7.27; *p* < 0.001), and no significant between-group difference at pre-test (WG pre = 4.56; CG pre = 3.94). These results are presented in Figure 4 below.

All students (SG vs. WG): the pre–post by group interaction was not significant (F < 1; *p* = 0.96).

##### Verbal Fluency Test

**Figure 5:**
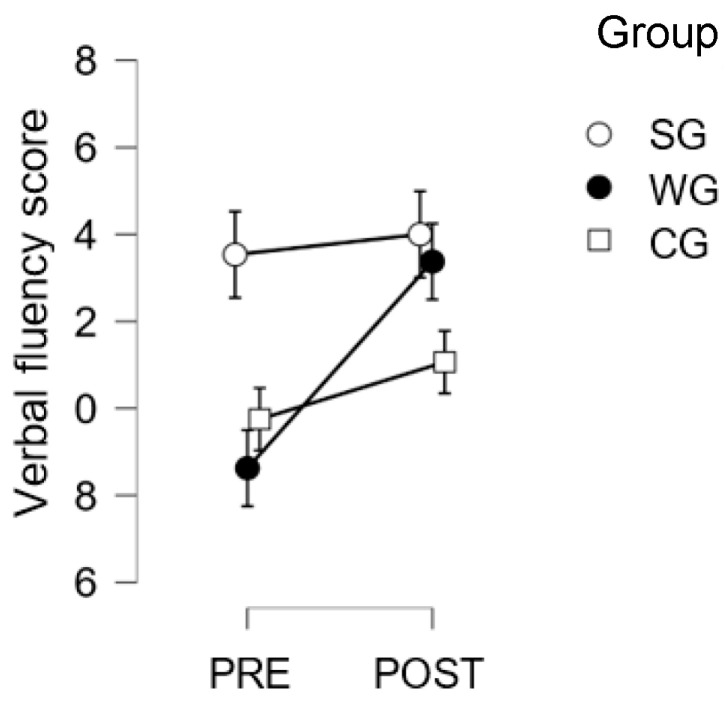
Verbal Fluency Test scores for 2nd graders, for the creative Writing Group, the Singing Group, and the Control Group, at pre- and post-test.

Second-grade students only (SG vs. WG vs. CG): the pre–post by group interaction was significant (F(2,44) = 3.46; *p* = 0.04; η2= 0.047), with a significant improvement in the writing group (pre = 8.63; post = 13.38; *p* < 0.004), as well as a significant difference at pre-test, where the singing group scored higher (pre = 13.5) than the writing group (*p* = 0.009; cf. Figure 5).

**Figure 6:**
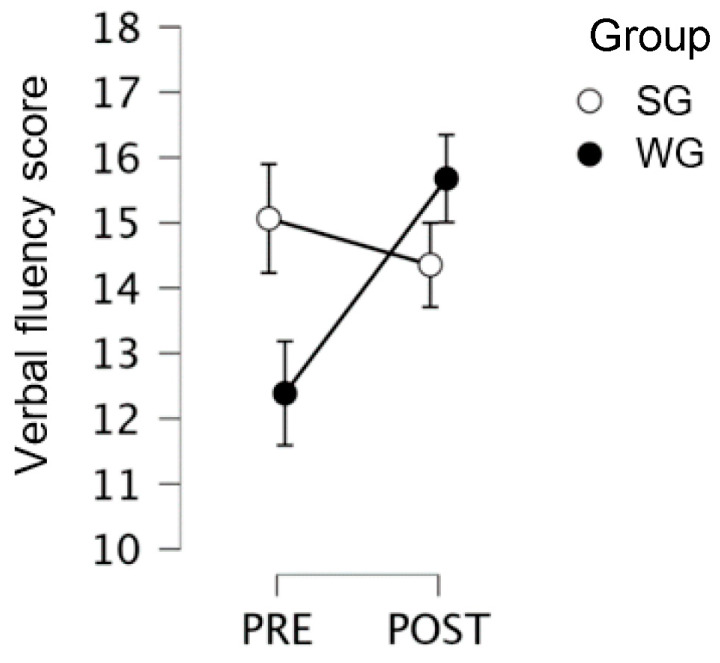
Verbal Fluency Test scores for 2nd, 4th, and 5th graders, for the creative Writing Group, the Singing Group, and the Control Group, at pre- and post-test.

All students: the pre–post by group interaction was significant (F(1,60) = 4.19; *p* = 0.045; η2 = 0.006), with significant improvement in the writing group (pre = 12.69; post = 15.81), *p* = 0.007) and no significant improvement in the SG (pre = 15; post = 14.35; no significant difference between the two SGs and WGs at pre-tests), as shown in Figure 6.

##### Cluster Analysis

Cluster analyses for the Alouette Reading Test showed that for the group of students with the highest levels of improvement, 46.3% were from the SG, 40.7% from the WG, and 13% from the CG (chi-square test: X^2^(2,80) = 5.78; *p* = 0.055). In other words, 78.1% of the students in the singing group were part of the cluster representing those who had made the most progress (cf. Table 1). For the Verbal Fluency Test, 51.1% of the cluster with the largest improvement came from the WG, 26.7% from the SG, and 22.2% from the CG (chi-square test: X^2^(2,80) = 8.0; *p* = 0.018). Finally, for the Similarities Test, the cluster analyses showed that 68.8% of children in the WG showed a large improvement, compared to only 53.1% in the SG and 43.8% in the CG (cf. Table 2). However, these between-group differences did not reach significance (X^2^(2,80) = 3.15; *p* = 0.207).

## 4. Discussion

The objective of this paper was to measure the impact of singing and creative writing activities, which were carried out every morning for 20 min over the school year, on the cognitive development of children from low socioeconomic backgrounds. To that end, eighty 2nd, 4th, and 5th graders took part in this study, and were included either in a Singing group (SG), a Writing group (WG), or in neither of these two groups (Control group—CG). We tested all of these children on a set of cognitive measures evaluating, among other things, language (oral and written: reading, phonological awareness, word dictation, vocabulary, verbal fluency), attention (both auditory and sustained), working memory, and creativity, at the beginning and the end of the school year, before and after the workshops were carried out.

First, the results showed that, overall, the children improved on most of the abilities evaluated (main effect of pre vs. post activity). In the eight months between the pre- and post-tests, students showed a level of progression in line with normal childhood development. However, there was one test that showed no improvement between the beginning and the end of the year, the Faux Pas Test. As described above, in this test, children are read short stories and are asked to identify if a person has said something they should not have said. To be successful, children must simultaneously imagine two states of mind: that of the person who unknowingly commits the faux pas and that of the person who is the victim of the faux pas [61]. For this, we used a French translation, which, although it has been widely used, has not yet been sampled on a French population, which may explain the lack of progression. Furthermore, this test is slightly outdated and no longer corresponds to the children’s reality. Therefore, it would have been appropriate to add a general comprehension test, such as the ELO, to evaluate oral comprehension skills and to serve as a control variable. For future research, a prisoner’s dilemma test, in which two children must either choose to cooperate for their collective interest or to “betray” one another for individual benefit, could be better suited for assessing pro-social behavior [62].

In addition, before demonstrating potential transfer effects on cognitive and language skills, it is important to show that the workshops had their expected effects, leading to significant improvements of the skills students were trained in. To measure students’ singing progress, we used two subtests adapted from the MBEA (Montreal Battery of Evaluation of Amusia), in which children heard two successive musical phrases and had to determine whether or not they were identical in terms of rhythm or melody. Initially used to measure the loss of ordinary musical skills due to brain damage or abnormalities, this battery is a validated and widely used tool under “normal” conditions as well. However, while it has been used extensively in studies involving instrumental practice, it has not been used to measure the effects of singing practice, which, as we have noted, have not been widely studied until now. Obviously, the lack of specific progression of the SG leads us to believe that this battery is not a good tool for measuring singing practice, for which it would be more beneficial to measure production rather than perception aspects. As the study of singing practice is still an underdeveloped topic, the few studies that focus on this skill either do not measure the evolution of singing skills on their own [62], or create their own tools to do so (Jungbluth, A., and Hafen, R. (2005), Musik-Screening für Kinder [Unpublished test material] cited in Degé et al. [17]). Here, we also created unique methods of measurement through our use of juries, made up of professional singers and writers, who assessed the children before and after their participation in the workshops. The results from these evaluations for the singing group showed progress on three criteria, namely focus (posture and concentration), the capacity to sing in unison, and rhythmic accuracy. As there is, to our knowledge, no standardized scale for assessing singing ability, an interesting challenge in the future would be to develop standardized tests for this purpose.

For creative writing groups, the results of the psychometric tests we used did not show significant progress in creativity (EPoC Test) or in handwriting skills (BHK Test). For the latter skill, the lack of progress is not completely surprising based on the fact that the children came from disadvantaged backgrounds, and such groups have been known to show major handwriting difficulties at the beginning of 2nd grade and even later, due to a lack of automation of the function [63,64]. Thus, the workshops consisted largely of oral reflection and imagination tasks, rather than focusing systematically on written work. However, we had expected an improvement in creative capacities, which should be captured by the EPoC Test, but our results did not align with this. One of the most important questions regarding creativity is whether it can be truly and effectively measured. The scientific study of creativity requires the construction of sensitive, reliable, and valid assessment instruments [65]. In practice, the EPoC Test has some limitations, especially in its scoring method. For some items, scoring is purely quantitative, such as for production, where only the number of productions made by children is counted, and their quality is ignored. Thus, children who create multiple repetitive drawings will receive a higher creativity score, and thus could inaccurately be considered more creative, than children who produce fewer, but more intricate drawings. In future studies, it will be necessary to adapt the scoring of this test, or to choose other, more relevant creativity tests (e.g., Torrance Test of Creative Thinking (TTCT), [66]). Regarding the students’ evaluations by the external jury, we saw that the children were better at writing in a given style after the workshops, but that the speed at which they started a writing task decreased. Nonetheless, there are various aspects that could impact the speed of a creative production, other than the creativity itself, such as a careless way of responding rather than a careful one [67,68].

As for the transfer effects on other cognitive and language skills, the results showed a significant progression for both the singing and writing groups on the Alouette Reading Test. Furthermore, cluster analyses showed that the group of children who had made the most progress on this test was made up of almost 80% of children from the singing group. Nonetheless, we assess these results cautiously as our effect size was quite small. The Alouette Reading Test is classically used to assess reading skills, specifically speed and accuracy, by requiring students to read a nonsense text aloud. Concerning the singing group, these results would largely corroborate those observed for the effects of musical practice on reading [69,70], which can be explained by transfer effects at different levels.

Musical practice, such as singing, improves pitch processing not only in music, but also in speech [9,10]. This finding argues for a close link between basic auditory perception skills and reading skills. In other studies involving the learning of new words, it has been shown that musically trained children and young adults outperform participants with no musical expertise. This advantage is manifested both at the behavioral and cerebral levels, pointing to a neural facilitation of access to lexical-semantic representations following musical training. Finally, language and music are closely related to higher-level processes such as attention and working memory [70], and many studies have shown that musical practice, which requires participants to focus their attention on sounds, or to remember musical pieces, improves these two skills, which are also largely involved in reading.

In line with this, our results also showed a significant improvement for children in the singing group on the Symbol Search Test. This test consists of identifying visual symbols as quickly as possible and is taken to reflect the speed of information processing and the ability to act under time pressure in an attentional task. These results are thus in agreement with the literature, which has previously illustrated an improvement on this test, and more generally, on attentional abilities, following musical training (e.g., [46]). Thus, for the first time to our knowledge, our study shows that, just as musical training has been shown to do, the practice of choral singing improves students’ capacities for focused attention and concentration. Regarding the performance of children who practiced creative writing, our results showed a significant improvement compared to the singing and control groups in the Alouette Reading Test, as well as in the Similarities Test and the Verbal Fluency Test. As a reminder, in the Similarities Test, children were asked to explain the similarities between two words or concepts that are related. This test measures concept elaboration and abstraction skills. Meanwhile, the general objective of the Verbal Fluency Test is to verify the students’ ability to access their lexical repertoire by generating as many words as possible in one minute that either start with a given letter (spelling fluency) or belong to a given semantic category (e.g., furniture). Thus, students’ improvement on these tests was expected, given that the creative writing workshop allowed children to learn new words, to use them within different contexts based on their various meanings, and to improve their generation of ideas and associations between them [71]. Moreover, the content of the creative writing workshops regularly comprised of reading and discussing the texts produced, which would also explain the improvement on the Alouette Reading Test. Finally, these results align with the previous literature, which has shown a positive relationship between reading skills and creative writing ability [72,73].

The results of this study have implications for educational practice. Indeed, they are quite relevant given the current reality of school difficulties within the French education system, particularly in terms of reading skills [2]. Given that singing and creative interventions had a positive impact on the children’s reading performance, this clearly indicates that these artistic methods could greatly contribute to the acquisition and improvement of reading. Moreover, these techniques could also help foster the development of critical thinking and mental flexibility.

In addition, even if these specific results were not conclusive in our study, some previous studies have shown the impact of singing on pro-social skills [74]. In our study, this was pointed out by the teachers, who told us, for example, that for the first time, girls and boys were playing football together in the schoolyard. Thus, given the social component involved in both artistic practices, and particularly singing, which requires synchronization, they could be an excellent way to practice and develop interpersonal and communication skills within the classroom. However, it is important to consider the current reality of how little cognitive sciences truly influence educational practices and guidelines [75]. Findings from said field are often not prioritized, given that there is a fixed scheduled program for the teachers to follow. Nonetheless, our results show how much the children could directly benefit from these types of artistic methods. Accordingly, integrating these techniques into the curricula could greatly enhance teaching practices, contributing to a more holistic educational approach.

Although this study is innovative and quite relevant within an educational context, some potential limitations should be highlighted. In our study, we had chosen a population located in a difficult and disadvantaged neighborhood of Marseille. Firstly, because the literature has shown that these children in difficulty benefit the most from these type of workshops. Secondly, beyond the potential cognitive issues, because these children have very little access to interventions of this type due to financial or logistical reasons. Therefore, these workshops give them an opportunity to broaden their minds, which they would not have had in their usual daily life. Our results show that these children are sensitive to these types of interventions and they progress more than children who have not taken part in these workshops. However, we do not know whether the results would be as relevant for children coming from more privileged backgrounds, thus we cannot extend our conclusions to this latter population.

Furthermore, this is a field experiment. That entails various advantages, but also some limitations. In our case, the participants could not be allocated in a perfectly randomized-controlled way to the different groups (singing, creative writing, control), which is the most scientifically rigorous method for evaluating the effectiveness of interventions. Indeed, it would have been logistically impossible to extract a few children from each class every morning to have them engage in one workshop or the other. Accordingly, there could be a “teacher effect”, which would mean that a given class could perform better partly because the teacher is more motivated and dynamic (this could be taken into account in the future using multi-level statistical analyses). Moreover, as previously mentioned, some of the tests were not correctly selected, given that they were either slightly outdated, translated into French but not sampled to the French population, or simply poorly designed to specifically measure what we needed. For future research, these tests will be likely replaced by more appropriate alternatives. Another limitation was that we tested fewer children than expected due to the COVID-19 pandemic. It is still a decent sample size for a field experiment; however, it limits the scope of our conclusions. Finally, as we pointed out before, the effect sizes observed are small to medium, so further studies of this type will be needed in the future to confirm our results.

## 5. Conclusions

The aim of this study was to measure the impact of choral singing and creative writing workshops on the cognitive and linguistic development of children from disadvantaged areas of Marseille. The originality of this research lies in the fact that these workshops were performed daily, as well as in their content, which until now has not been widely studied in the literature, as compared with activities such as instrumental practice. Here, children were involved in a longitudinal protocol, where they were tested at the beginning and end of the school year. Despite working with a smaller sample size than expected, mainly because of complications related to the COVID-19 pandemic, the workshops were able to continue throughout the year, and the results presented here are promising. They show that in eight months of daily choral singing, children significantly improved their reading and attentional skills, and that those who practiced creative writing improved their vocabulary and reading skills as well. These results highlight new ways of providing support for children from disadvantaged backgrounds, and we thus encourage teachers to incorporate these types of artistic practices into their daily teachings. This is especially true of singing, which, unlike other musical practices, does not require an instrument, and is therefore less logistically and economically costly. Furthermore, compared with other musical practices it is an activity carried out collectively, which could greatly benefit social skills. It would be relevant to further study the said impact in the future.

Despite the fact that the links between creative writing and cognitive skills or academic performance are still inconclusive and understudied, the results obtained here are encouraging and set a precedent for the use of creativity exercises in the classroom. The value of this type of practice lies in getting students to explore oral language in a different way, and to share their productions in a safe environment, thus enabling the construction of new knowledge. Moreover, our results emphasize the importance of continuing the promotion of creativity in the academic curricula, to benefit the students’ cognition and academic achievements, especially considering that creativity has been appointed a key skill for future individual and social success [76].

## Figures and Tables

**Table 1:** Contingency tables for the Alouette Reading Test. The cluster “1” is composed of the children with the highest rate of progress.

Contingency Tables					
		Workshop			
Alouette Reading Test		SG	WG	CG	Total
1	Observed	25	22	7	54
	% within row	46.3%	40.7%	13.0%	100.0%
	% within column	78.1%	68.8%	43.8%	67.5%
2	Observed	7	10	9	26
	% within row	26.9%	38.5%	34.6%	100.0%
	% within column	21.9%	31.3%	56.3%	32.5%
Total	Observed	32	32	16	80
	% within row	40.0%	40.0%	20.0%	100.0%
	% within column	100.0%	100.0%	100.0%	100.0%

**Table 2:** Contingency tables for the Verbal Fluency Test. The cluster “1” is composed of the children with the highest rate of progress.

Contingency Tables					
		Workshop			
Verbal Fluency Test		SG	WG	CG	Total
1	Observed	12	23	10	45
	% within row	26.7%	51.1%	22.2%	100.0%
	% within column	37.5%	71.9%	62.5%	56.3%
2	Observed	20	9	6	35
	% within row	57.1%	25.7%	17.1%	100.0%
	% within column	62.5%	28.1%	37.5%	43.8%
Total	Observed	32	32	16	80
	% within row	40.0%	40.0%	20.0%	100.0%
	% within column	100.0%	100.0%	100.0%	100.0%

## Data Availability

The data presented in this study are available on request from the corresponding author. Data sharing is not applicable to this article.

## Appendix A

**Table A1:** Table with the characteristics of the children.

Group Activity	2nd			4th			5th	
		Singing	Writing	Control	Singing	Writing	Singing	Writing
		16	16	16	8	8	8	8
Average Age	months	84	84.8	84	108	108	120	120
	*t* test (*p* value)	S vs. W	W vs. C	S vs. C	1		1	
		0.33	0.59	1				
Gender	Girls	6	4	8	4	4	4	4
	Boys	10	12	8	4	4	4	4
	Chi^2^ (*p* value)	S vs. W	W vs. C	S vs. C	1		1	
		0.45	0.14	0.48				
Handedness	R	14	11	12	8	8	8	8
	L	2	5	4	0	0	0	0
Extracurricular Activity (%)	Total	56.3	56.3	37.5	62.5	37.5	62.5	87.5
	Art	12.5	6.3	6.3	0	25	0	12.5
	Sport	43.8	56.3	31.25	62.5	25	62.5	75
	Music	6.3	0	0	12.5	0	0	37.5
Learning Disability (%)	Dyslexia	12.5	12.5	0	12.5	12.5	12.5	12.5
	Dyspraxia	12.5	0	0	0	0	0	0
	Other (s)	12.5	0	18.8	12.5	0	0	0

## References

[1] Bulletin Officiel [BO] du 12 Janvier 2023. Note de Service du 10-1-2023 (NOR: MENE2300947N). Savoirs Fondamentaux—Renforcer la Maîtrise des Savoirs Fondamentaux des Élèves en CM1, CM2 et 6e (Cycle 3) Pour Faciliter Leur Entrée au Collège. https://www.education.gouv.fr/bo/23/Hebdo2/MENE2300947N.htm.

[2] (2016). CNESCO Note D’actualité. Ce Que Les Enquêtes Internationales (PISA, TIMSS) Peuvent Nous Dire de L’état de L’école Française. https://www.cnesco.fr/wp-content/uploads/2016/12/161206_Note_PISA.pdf.

[3] OECD (2019). Résultats du PISA 2018 (Volume I): Savoirs et Savoir-Faire des Elèves.

[4] Frey A., Sappey-Marinier A. (2018). La Musique Comme Vecteur de Développement Langagier: Effet d’un Entraînement Musical sur les Compétences Langagières Chez des Enfants de CE2. Ressources.

[5] Bigand E., Tillmann B. (2021). Near and far transfer: Is music special?. Mem. Cogn..

[6] Román-Caballero R., Vadillo M.A., Trainor L.J., Lupiáñez J. (2022). Please don’t stop the music: A meta-analysis of the cognitive and academic benefits of instrumental musical training in childhood and adolescence. Educ. Res. Rev..

[7] Sala G., Gobet F. (2017). When the music’s over. Does music skill transfer to children’s and young adolescents’ cognitive and academic skills? A meta-analysis. Educ. Res. Rev..

[8] Sala G., Gobet F. (2017). Does Far Transfer Exist? Negative Evidence from Chess, Music, and Working Memory Training. Curr. Dir. Psychol. Sci..

[9] Gordon R.L., Schön D., Magne C., Astésano C., Besson M. (2010). Words and Melody Are Intertwined in Perception of Sung Words: EEG and Behavioral Evidence. PLoS ONE.

[10] Schön D., Gordon R., Campagne A., Magne C., Astésano C., Anton J.-L., Besson M. (2010). Similar cerebral networks in language, music and song perception. NeuroImage.

[11] Engel E. (2020). The Effect of Songs and Chants with Words on Phonological Awareness in Early Childhood. Doctoral Dissertation.

[12] Walton P. (2014). Using Singing and Movement to Teach Pre-reading Skills and Word Reading to Kindergarten Children: An Exploratory Study. Lang. Lit..

[13] Christiner M., Reiterer S.M. (2013). Song and speech: Examining the link between singing talent and speech imitation ability. Front. Psychol..

[14] Christiner M., Reiterer S.M. (2015). A Mozart is not a Pavarotti: Singers outperform instrumentalists on foreign accent imitation. Front. Hum. Neurosci..

[15] Christiner M., Rüdegger S., Reiterer S.M. (2018). Sing Chinese and tap Tagalog? Predicting individual differences in musical and phonetic aptitude using language families differing by sound-typology. Int. J. Multiling..

[16] Christiner M., Reiterer S.M. (2018). Early influence of musical abilities and working memory on speech imitation abilities: Study with pre-school children. Brain Sci..

[17] Degé F., Müllensiefen D., Schwarzer G. (2020). Singing abilities and phonological awareness in 9- to 12-Year-Old children. Yearb. Music. Psychol..

[18] Demos A.P., Chaffin R., Begosh K.T., Daniels J.R., Marsh K.L. (2012). Rocking to the beat: Effects of music and partner’s movements on spontaneous interpersonal coordination. J. Exp. Psychol. Gen..

[19] Müller V., Lindenberger U. (2011). Cardiac and Respiratory Patterns Synchronize between Persons during Choir Singing. PLoS ONE.

[20] Hove M.J., Risen J.L. (2009). It’s All in the Timing: Interpersonal Synchrony Increases Affiliation. Soc. Cogn..

[21] Chobert J., François C., Velay J.-L., Besson M. (2014). Twelve Months of Active Musical Training in 8- to 10-Year-Old Children Enhances the Preattentive Processing of Syllabic Duration and Voice Onset Time. Cereb. Cortex.

[22] Schellenberg E.G. (2004). Music Lessons Enhance IQ. Psychol. Sci..

[23] Runco M.A., Jaeger G.J. (2012). The Standard Definition of Creativity. Creat. Res. J..

[24] Colin T. (2017). Analyzing Ambiguity in the Standard Definition of Creativity. AVANT J. Philos. Vanguard.

[25] Abraham A., Rutter B., Hermann C. (2021). Conceptual expansion via novel metaphor processing: An ERP replication and extension study examining individual differences in creativity. Brain Lang..

[26] Cremin T., Chappell K., Craft A. (2013). Reciprocity between narrative, questioning and imagination in the early and primary years: Examining the role of narrative in possibility thinking. Think. Ski. Creat..

[27] Lucas B., Greany T. (2000). Schools in the learning age. Campaign for Learning.

[28] Kautz T., Heckman J., Diris R., Weel B., Borghans L. (2014). Fostering and Measuring Skills: Improving Cognitiveand Non-Cognitive Skills to Promote Lifetime Success (OECD Education Working Papers, No. 110).

[29] Lucas B. (2016). A Five-Dimensional Model of Creativity and its Assessment in Schools. Appl. Meas. Educ..

[30] Ai X. (1999). Creativity and Academic Achievement: An Investigation of Gender Differences. Creat. Res. J..

[31] Balgiu B.A., Adîr V. (2014). Creativity tasks and academic achievement. A study on Romanian Politehnica Undergraduate Students. Procedia-Soc. Behav. Sci..

[32] Berlin N., Tavani J.-L., Beasançon M. (2016). An exploratory study of creativity, personality and schooling achievement. Educ. Econ..

[33] Vijetha P., Jangaiah C. (2010). Intelligence, Creative Thinking Abilities and Academic Achievement of Children with Hearing Impairment-A Correlation Study. J. All India Inst. Speech Hear..

[34] Tan M., Mourgues C., Bolden D.S., Grigorenko E.L. (2014). Making numbers come to life: Two scoring methods for creativity in Aurora's Cartoon Numbers. J. Creat. Behav..

[35] Gajda A. (2016). The relationship between school achievement and creativity at different educational stages. Think. Ski. Creat..

[36] Castillo-Vergara M., Alvarez-Marin A., Placencio-Hidalgo D. (2018). A bibliometric analysis of creativity in the field of business economics. J. Bus. Res..

[37] Yang J., Zhao X. (2021). The effect of creative thinking on academic performance: Mechanisms, heterogeneity, and implication. Think. Ski. Creat..

[38] Sahbaz N.K., Duran G. (2011). The efficiency of cluster method in improving the creative writing skill of 6th grade students of primary school. Educ. Res. Rev..

[39] Temizkan M. (2011). The Effect of Creative Writing Activities on the Story Writing Skill. Educ. Sci. Theory Pract..

[40] Essex C. (1996). Teaching Creative Writing in the Elementary School.

[41] Tompkins G.E. (1982). Seven reasons why children should write stories. Lang. Arts.

[42] Kleber B.A., Zarate J.M. (2014). The Neuroscience of Singing. The Oxford Handbook of Singing.

[43] Zhou K. (2018). What cognitive neuroscience tells us about creativity education: A literature review. Glob. Educ. Rev..

[44] Bruner R.F. (2001). Repetition Is the First Principle of All Learning. https://www.researchgate.net/publication/228318502_Repetition_is_the_First_Principle_of_All_Learning.

[45] Paquet A. (2017). Effets de la Pratique Musicale sur le Rappel Actif Auditif de L’enfant: Une Étude de Potentiels Évoqués. https://archipel.uqam.ca/10908/.

[46] Barbaroux M., Dittinger E., Besson M. (2019). Music training with Démos program positively influences cognitive functions in children from low socio-economic backgrounds. PLoS ONE.

[47] Lubart T., Besançon M., Barbot B. (2011). EPoC—Mesure du Potentiel Créatif des Enfants.

[48] Peretz I., Champod A.S., Hyde K. (2003). Varieties of musical disorders. The Montreal Battery of Evaluation of Amusia. Ann. N. Y. Acad. Sci..

[49] Korman M., Kirk S., Kemp S. (2012). NEPSY II Bilan Neuropsychologique de L’enfant.

[50] Cardebat D., Doyon B., Puel M., Goulet P., Joanette Y. (1990). Évocation lexicale et sémantique chez les sujets normaux. Performance dynamique de production en fonction du sexe, de l’âge et du niveau d’étude. Acta Neurol. Belg..

[51] Wechsler D. (2016). WISC V—Échelle D’intelligence de Wechsler Pour Enfants et Adolescents.

[52] Raven J.C., Court J.H. (1998). Manual for Raven’s Progressive Matrices and Vocabulary Scales.

[53] Wiig E., Semel E., Secord W.A. (2019). CELF 5—Batterie D’évaluation des Fonctions Langagières et de Communication.

[54] Jacquier-Roux M., Valdois S., Zorman M., Lequette C., Pouget G. (2005). ODEDYS: Outil de DÉpistage des DYSlexies Version 2.

[55] Lafavrais P. (2005). Alouette-R, Les Éditions du Centre de Psychologie Appliquée.

[56] Garrigues E., Gobillot C. (2013). Traduction et adaptation du Faux Pas Test et faits cliniques. Mémoire Pour le Certificat de Capacité D’orthophonie.

[57] Brickenkamp R., Liepman D., Schmidt L. (2015). D2-R Test D’attention Concentrée Révisé.

[58] Charles M., Soppelsa R., Albaret J.-M. (2004). BHK, Échelle D’évaluation Rapide de L’écriture Chez L’enfant, EAP édition.

[59] JASP Team (2002). JASP (Version 0.16.2). https://jasp-stats.org/.

[60] (2021). The Jamovi Project, v.1. http://www.jamovi.org.

[61] Baron-Cohen S., O’Riordan M., Stone V., Jones R., Plaisted K. (1999). Recognition of Faux Pas by Normally Developing Children and Children with Asperger Syndrome or High-Functioning Autism. J. Autism Dev. Disord..

[62] Good A., Russo F.A. (2016). Singing Promotes Cooperation in a Diverse Group of Children. Soc. Psychol..

[63] Wicki W., Lichtsteiner S.H. (2018). Improvement of handwriting automaticity among children treated for graphomotor difficulties over a period of six months. J. Occup. Ther. Sch. Early Interv..

[64] Potter C. (2017). Developing Automaticity in Children with Learning Disabilities: A Functional Perspective Part One: Theory and Assessment. Learning Disabilities—An International Perspective.

[65] Lubart T., Mouchiroud C., Tordjman S., Zenasni F. (2015). Psychologie de la Créativité.

[66] Torrance E. (1966). Torrance Tests of Creative Thinking.

[67] Bowling N.A., Huang J.L., Bragg C.B., Khazon S., Liu M., Blackmore C.E. (2016). Who cares and who is careless? Insufficient effort responding as a reflection of respondent personality. J. Pers. Soc. Psychol..

[68] Huang J.L., Curran P.G., Keeney J., Poposki E.M., DeShon R.P. (2012). Detecting and Deterring Insufficient Effort Responding to Surveys. J. Bus. Psychol..

[69] Moreno S., Marques C., Santos A., Santos M., Castro S.L., Besson M. (2009). Musical Training Influences Linguistic Abilities in 8-Year-Old Children: More Evidence for Brain Plasticity. Cereb. Cortex.

[70] Besson M., Chobert J., Marie C. (2011). Language and Music in the Musician Brain. Lang. Linguist. Compass.

[71] Akdal D., Şahin A. (2014). The Effects of Intertextual Reading Approach on the Development of Creative Writing Skills. Eurasian J. Educ. Res..

[72] Broekkamp H., Janssen T., Bergh H.V.D. (2009). Is There a Relationship between Literature Reading and Creative Writing?. J. Creat. Behav..

[73] Razgatlıoğlu M., Education M.O.N., Ulusoy M. (2022). Gazi University the Effect of Activity-Based Poetry Studies on Reading Fluency and Creative Writing Skills. Int. J. Progress. Educ..

[74] Welch G.F., Himonides E., Saunders J., Papageorgi I., Sarazin M. (2014). Singing and social inclusion. Front. Psychol..

[75] Rittle-Johnson B., Star J.R., Durkin K. (2020). How Can Cognitive-Science Research Help Improve Education? The Case of Comparing Multiple Strategies to Improve Mathematics Learning and Teaching. Curr. Dir. Psychol. Sci..

[76] OECD (2019). Pisa 2021 Creative Thinking Framework.

